# Supplementary material for: A Global Screen for Assembly State Changes of the Mitotic Proteome by SEC-SWATH-MS
Source: Cell Syst. 2020 Feb 26;10(2):133–155.e6. doi: 10.1016/j.cels.2020.01.001 (PMC7042714; doi:10.1016/j.cels.2020.01.001)

P63167 | DYL1_HUMAN | DYNLL1 DLC1 DNCL1 DNCLC1 HDLC1
Monomer MW [kDa]: 10.366 Monomer expected elution fraction: 60

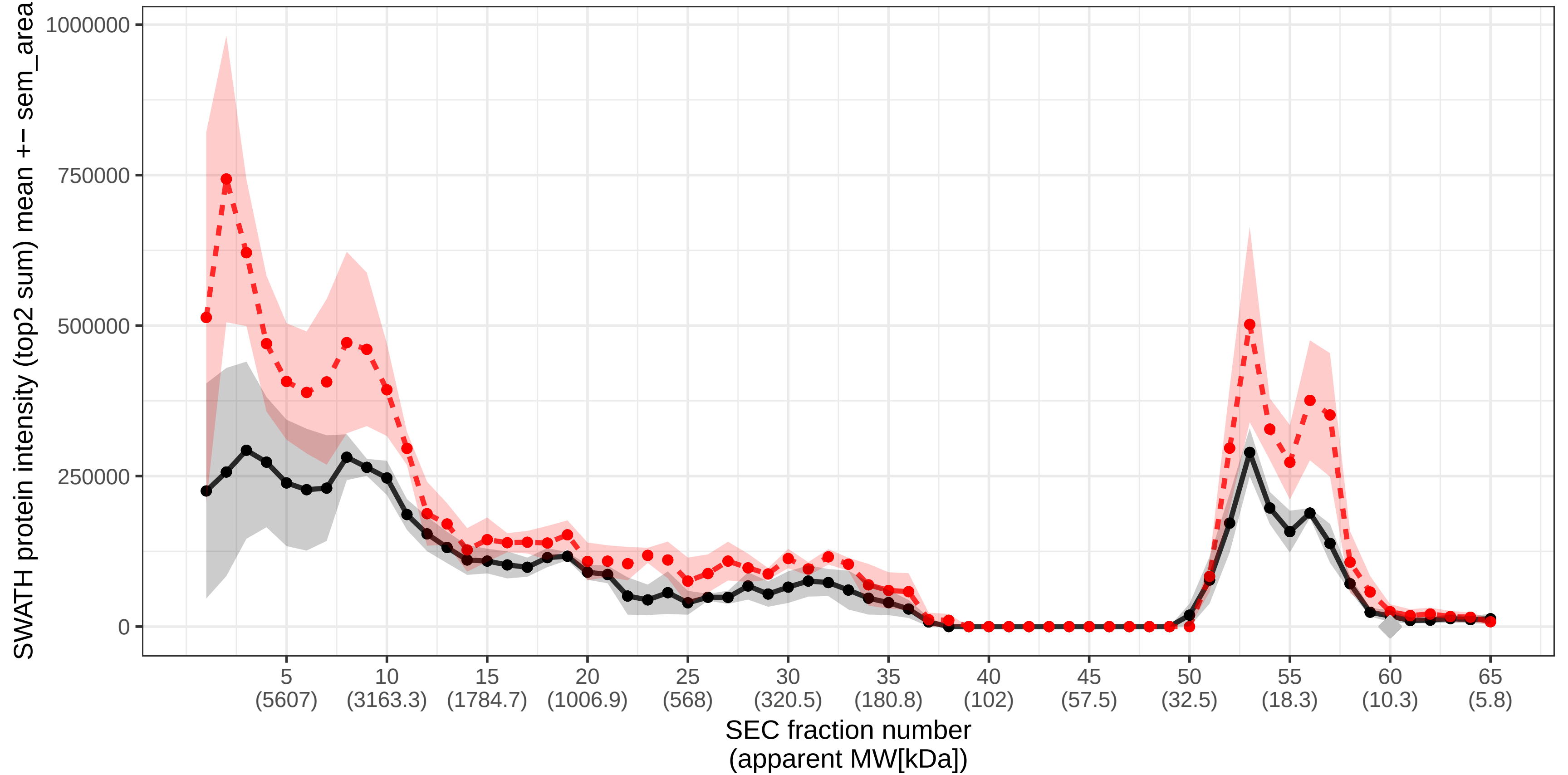

Supplement: Data S1. SEC-SWATH-MS Protein Chromatograms, Related to Figure 1 [file mmc6.zip › SECchrom_P63167_DYL1_HUMAN_DYNLL1_DLC1_DNCL1_DNCLC1_HDLC1.pdf]
